# Effect of Shell Growth on the Morphology of Polyvinyl Acetate/Polystyrene Inverted Core-Shell Latex Fabricated by Acrylonitrile Grafting

**DOI:** 10.3390/ma11122482

**Published:** 2018-12-06

**Authors:** Jiaxing Sun, Xiao Zhang, Long Bai, Zhiguo Li, Zhao Jia, Jiyou Gu

**Affiliations:** 1 College of Materials Science and Engineering, Northeast Forestry University, Key Laboratory of Bio-based Material Science and Technology, Ministry of Education, Harbin 150040, China; sunjiaxing0209@163.com (J.S.); zx2915@hotmail.com (X.Z.); wtunefu@163.com (Z.J.); 2 Department of Bioproducts and Biosystems, School of Chemical Engineering, Aalto University, P.O. Box 163000, FIN-00076 Aalto, 02150 Espoo, Finland; longbai2011@hotmail.com

**Keywords:** inverted core-shell particle, polyvinyl acetate, acrylonitrile grafting, shell growth, morphological evolution

## Abstract

A novel strategy for fabricating inverted core-shell structured latex particles was implemented to investigate the morphology and properties of polyvinyl acetate (PVAc)-based latex. In this study, active grafting points were synthesized onto the surface of PVAc latex cores via grafting acrylonitrile (AN) to obtain a controllable coating growth of the shell monomer, styrene (St). The effect of shell growth on the morphological evolvement was explored by tuning the time of shell monomer polymerization. Unique particle morphologies, transferring from “hawthorn” type, over “peeled pomegranate” type, to final “strawberry-like” type, were observed and verified by electron microscopy. The morphological structure of latex particles exerted a significant effect on the particle size, phase structure, and mechanical properties of the obtained emulsions. The water-resistance of PVAc-based latex was also evaluated by the water absorption of latex films. More importantly, the experimental results provided a reasonable support for the controlled growth of St monomer, that is, the self-nucleation of dispersive St monomer can be transformed to in-situ coating growth on the PVAc core surface depending on the AN-active grafting points. This fabricating approach provides a reference for dynamical design and control of the latex particle morphology.

## 1. Introduction

Core-shell structure was commonly used to design and prepare structured emulsion-based polymers owing to its special heterogeneous structure and stratification effect [1,2,3,4,5,6]. With the deepening of theoretical research and the enriching of the morphological structure, properties, and applications of core-shell latex, the concept of core-shell emulsion and its construction technology have been increasingly expanded [7]. Therefore, how to design and synthesize well-defined core-shell latex particles with a unique morphology, structure, and function attracts considerable attention in the field of colloid research [8]. Furthermore, the ability of core-shell structured latex particles to combine components showing different thermodynamic features in a single composite system provides a great opportunity for advanced materials and smart polymers with multi-functionalities [9,10]. The fabricating procedures for core-shell structured latex, such as multiple-stage emulsion polymerization [11,12], interfacial process [13], and heterocoagulated reaction [2], have been well reported. Among these approaches, emulsion polymerization was a facile, efficient, and cost-effective method. For emulsion-based core-shell, the latex particles with a fully covered surface are commonly formed from the inorganic/organic hybrid system [14,15], which limits the functional design and applications of obtained products. On the other hand, some special morphologies including strawberry- and sandwich-like can be successfully formed from a polymer-based core-shell system. In summary, the core factor for determining the morphology of structured particles can be attributed to whether the morphology of the latex particle attains thermodynamic equilibrium, which may be affected by the hydrophobicity of core and shell components, interfacial energy between different phases and other related factors. For an organic/organic system, the thermodynamic equilibrium ball-like morphology is easily obtained since the principle of minimum energy can be reached [16,17]. However, in a polymer-based system, besides the thermodynamics, many studies have shown that kinetics also play a key role in determining the morphology due to the more complicated evolving environment for polymer emulsion polymerization. Therefore, it is promising to generate more unique particle morphologies in a polymer-based emulsion polymerization process. As is well known, the properties of materials correspond with their basic units and how they assemble, leading to the fact that the composition, structure, and spatial configuration of the unit are greatly impacted. Hence, for the well-defined latex system, controlled morphology was an inevitable factor for revealing high-performance colloidal material. In our previous studies, the strategy for fabricating an inverted core-shell structure in colloidal particles with a specific morphology has proven to be very effective, especially for the multicomponent polymerization with a large difference among different components [11,18].

In this study, to overcome the inherent poor mechanical properties of polyvinyl acetate (PVAc), particularly wet strength, vinyl acetate (VAc) and styrene (St) were selected as the main components to design and fabricate PVAc/ Polystyrene (PS) latex particles with an inverted core-shell structure. The core-shell structured PVAc/PS particles might retain the excellent film-forming and adhesion properties of PVAc latex, in the meantime, the resistance of PVAc to water, heat, and creep can be improved by the embedded PS shells. However, it is well known that VAc and St do not copolymerize due to the unfavorable reactivity ratios and the difference between their kinetics (one polymerizes slowly, the other rapidly) and physical properties (one hydrophobic, the other hydrophilic; one with a high glass transition temperature, the other low) [19,20,21,22]. Therefore, a simple combination by co-polymerization VAc and St was difficult to achieve even in core-shell emulsion polymerization. In our previous study, latex particles with thermodynamic non-equilibrium inverted core-shell structured phase morphology were designed and prepared to obtain PVAc-based particles containing PVAc cores and PS shells. Recently, two approaches have been successfully developed, either by introducing functional active sites on PVAc cores to achieve a chemically grafted shell and to kinetically avoid the phase reversal in latex particles [11] or by fabricating a transition layer between the phases to reveal a physical barrier [12]. The construction of the transition layer and grafting sites could restrain the infiltration of St monomer and the free radicals into the core latex particles, thereby kinetically regulating the morphology to achieve thermodynamic non-equilibrium latex particles [23]. On the other hand, previous studies have only examined the final morphology of latex particles, but the dynamic evolvement of particle morphologies during the formation process remains unknown. Therefore, it is necessary to systematically study the formation process of the structured particles for fine tuning the preparation and high-performance application of PVAc latex.

Herein, an approach that adjusts the morphology of latex particles by controlling the degree of growth of the shell layer on the surface of PVAc cores was proposed. Specifically, acrylonitrile (AN) was used as the grafting monomer in this study to regulate the reactivity ratio and provide active growing points for the polymerization of St shell monomers. The effect of shell growth on the morphology of latex particles and its evolution behavior were studied by adjusting the shell polymerization time. This approach not only fabricates latex particles with a stable inverted core-shell structure but also enables a finely tunable system that can be generalized to other heterogeneous structured systems containing different constructing components.

## 2. Materials and Methods

### 2.1. Materials

Vinyl acetate (VAc) and Styrene (St) were purchased at reagent grade from Aladdin (Shanghai, China). The inhibitor component of the monomer was removed by vacuum distillation prior to polymerization. Acrylonitrile (AN) and a buffering agent, sodium bicarbonate (NaHCO_3_), were purchased from the Tianjin Fuchen Chemical Reagents Factory (Tianjin, China) at reagent grade. Initiator ammonium persulfate (APS) was purchased from Aladdin (Shanghai, China) at reagent grade. Emulsifiers, alkyl alcohol polyether sulfate sodium salt (PCA078) and alkyl ethoxy sulfosuccinate two sodium salt (PCA507), were purchased from Huntsman (Shanghai, China) surfactant Corporation at industrial grade. Deionized water was used throughout the experiments.

### 2.2. Latex Preparation

Recipes and reaction conditions for all the experiments were given in Table 1. The resulted two-stage inverted core-shell structured latexes were named as CS-x. The preparation method was the same as that reported by Zhang et al. [9] in a related study.

#### 2.2.1. Synthesis of Seed Latex

The temperature of the experiment was set to 60 °C. Deionized water, pH buffer (NaHCO_3_), and a mixture of anionic emulsifiers (PCA507 and PCA078) were mixed in a 500 mL four-neck flask and stirred (250 rpm) for at least 30 min. Then, a certain amount of VAc monomer (16% of the total monomer content) was added into the flask as the seed monomer. After stirring for 30 min, APS aqueous solution was added. The reaction mixture was heated up to 80 °C and the seed latex was thus obtained after 30 min reaction. It is worth noting that the seed latex prepared in this step will be suitable for all subsequent experiments.

#### 2.2.2. Synthesis of Core-Shell Structure Emulsion

The rest of the core monomer (VAc) was added dropwise (0.4 mL/min) into the seed latex within 120 min. Meanwhile, the aqueous solution of APS was added at a rate of one drop every 4 s. The polymerization was carried out for another 120 min at 80 °C. After which, the core latex was obtained. After that, AN monomer (2.0% of the total monomer content) was rapidly added (0.8 mL/min) into the reaction mixture within 2 min. Upon adding all the AN monomer, St monomer was immediately added dropwise (0.2 mL/min) into the core latex within 120 min. The reaction temperature at this stage was 80 °C. Further, samples were taken at the time when the shell monomer polymerization time was 30, 60, 90, and 120 min. Finally, the reaction system was heated to 85 °C for 30 min after the completion of St addition. 

### 2.3. Characterization

Fourier transform infrared spectroscopy (FTIR) was performed at the film-air interfaces with a Nicolet 6700 FTIR spectrometer (Thermo Fisher Scientific, St, Louis, MO, USA). All samples were mixed and ground with spectroscopic-grade potassium bromide and were scanned over range of 400–4000 cm^−1^. The latex film sample was mixed and ground with the appropriate amount of spectroscopic-grade potassium bromide (KBr) powder and pressed into a translucent sheet for testing. Spectral outputs were recorded as a function of wavenumber.

The glass transition temperatures (*T_g_*) of latex film samples were characterized by a differential scanning calorimeter (DSC-204, Netzsch, Germany) under a nitrogen atmosphere. The samples were first heated from room temperature to 180 °C and then cooled down to −30 °C with the rate of 20 K/min and maintained at −30 °C for 3 min. The samples were once again heated up to 180 °C with a heating rate of 10 K/min. The *T_g_* was defined as the mid-point of the change in the heat capacity on the heat flow and temperature plots of the second scan. The weight of the sample was about 5–8 mg.

The particle size and distribution of latex samples were measured with a dynamic light scattering (DLS, Zeta PALS Nano DLS, Malvern panalytical Ltd., Malvern, UK). The samples of emulsions were diluted with deionized water to adjust the solid content to about 0.01 wt% and placed in a clean quartz cuvette after ultrasonic shock before characterization. 

The surface morphology of samples was observed by a scanning electron microscope (SEM) (QuanTa-200, FEI Ltd., HL, USA) and a transmission electron microscope (TEM) (Hitachi-7650, Hitachi Co., Ltd., Tokyo, Japan), respectively. For SEM, latex particles were diluted to below 0.01 wt% and then a drop of diluent latex dispersion was placed on an aluminum foil to dry at ambient temperature. The coated aluminum foil was attached to the sample table by a conductive adhesive and then coated with a layer of gold–palladium before observation. For TEM, latexes were diluted to below 0.01 wt% and then a drop of the diluted latex was placed on a copper grid (usually 300 mesh). Then, the grids supporting the particles were placed in ruthenium tetraoxide (RuO_4_) vapor for a certain time. Micrographs were subsequently scanned after staining. In the micrographs, the PS-based phase appeared as dark domains and the PVAc-based phase appeared as bright domains.

A dynamic mechanical analyzer (DMA-242, Netzsh, Germany) was used to measure the temperature dependence of the storage moduli (E′) and the loss factor (tan δ), operating in tensile mode under isochronal conditions at the frequency of 1 Hz. The film sample was approximately 20 mm long, 5 mm wide, and 400 μm thick. The viscoelastic spectrum was recorded from −20 to 120 °C at a heating rate of 5 °C/min.

The water absorption of the latex film was the mass ratio of the difference between the mass before (*m*_1_) and after immersion of the latex film for 24 h (*m*_2_) and the mass before the specimen was immersed. The samples size was 10 mm × 20 mm × 0.4 mm, and the samples were immersed in water at 20 ± 1 °C and pH = 7 ± 1 for 24 h ± 15 min after being marked and weighed (to the nearest 0.0001 g). After removing the samples, their surface was dried with filter paper, weighed (to the nearest 0.0001 g), and recorded. The water absorption (*W*, expressed by percentage (%)) of the latex film was calculated as Equation (1).

(1) $$W=\frac{m_{2}-m_{1}}{m_{1}}\times 100\%$$

## 3. Results and Discussion

### 3.1. Characterization of Structured Latexes

Core-shell latex, as one of the special structured emulsion systems, displayed two or more obvious phase-separated structures, meaning that the cores and shells were synthesized by different components [24]. Considering that the inverted core-shell structure was at a thermodynamic non-equilibrium when compared with the traditional core-shell structure, a particular fabricating approach is necessary. Therefore, a functional monomer, acrylonitrile (AN), was employed to form the grafting structure on the surface of the monitored PVAc cores, providing active grafting sites to initiate the polymerization of St monomer. The chemical bond formed from AN-grafting points can build a forceful obstacle at the core and shell interfaces, and thus the turnover of the inverted core-shell structure may be greatly suppressed. In order to effectively control the particle size, promote monomer conversion, and further reveal the morphological evolution of the particles, semi-continuous seeded emulsion polymerization was used throughout the emulsion polymerization and St monomer was added in a “starved feeding” manner, with which the reaction rate of St was considered slower or equal to the feeding rate of the monomer. The coating growth of St monomer on the core surface to form strawberry-like latex particles was monitored and the characteristics of the obtained PVAc-AN/PS inverted core-shell structured latexes were prepared in the condition of 2% AN and a 7:3 core-to-shell ratio. 

**Figure 1:**
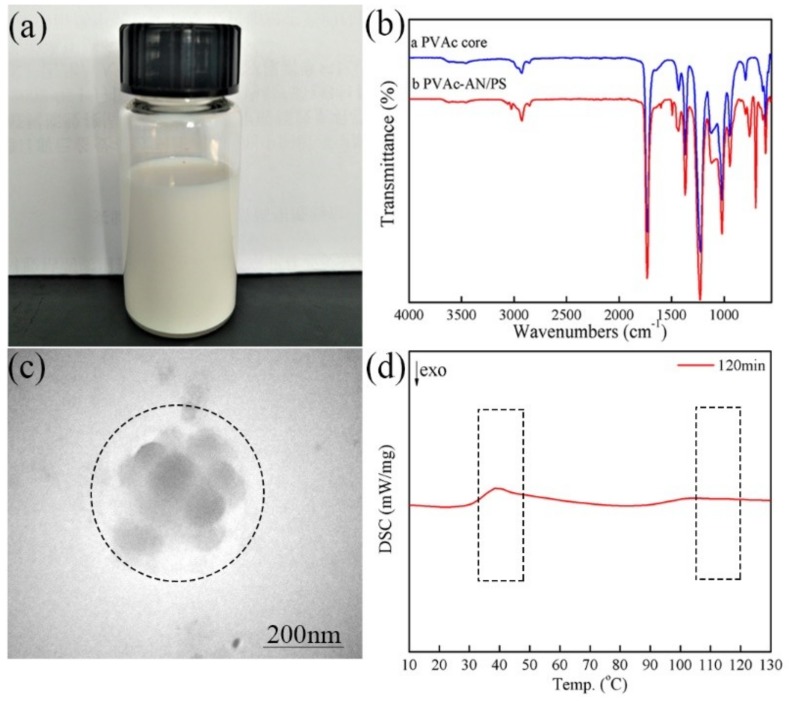
PVAc-AN/PS latex: (**a**) visual appearance of emulsion sample; (**b**) FTIR spectra; (**c**) TEM image; (**d**) DSC curve.

The visual appearance, FTIR spectra, TEM image, and DSC curve of the corresponding emulsion sample are displayed in Figure 1a–d, respectively. In Figure 1a, it can be observed that the prepared emulsion was homogeneous and appeared as ivory-white. In the FTIR spectra, shown in Figure 1b, the characteristic peak relating to the –C=O stretching vibration of acetate groups was at 1735 cm^−1^. The peaks at 1237 cm^−1^ and 1020 cm^−1^ were assigned to the asymmetric and symmetric stretching mode of the C–O–C of the ester group. The out-of-plane bending vibration absorption peaks of –CH on the mono-substituted benzene skeleton appeared at 756 cm^−1^ and 697 cm^−1^, respectively, which is a characteristic absorption peak of aromatic hydrocarbons. The bending vibration absorption peaks of C=C double bonds on the benzene ring skeleton appeared at 1600 cm^−1^, 1494 cm^−1^, and 1440 cm^−1^, manifesting that PVAc, PS, and AN were all incorporated into latex particles. Particularly, a new absorption peak was identified at 2217 cm^−1^, which can be attributed to the characteristic absorption band of –CN from acrylonitrile (AN), further indicating the existence of AN in the latex particles. For the TEM image, shown in Figure 1c, obvious outer dark protuberances and bright internal domains were observed, which can be ascribed to the stained PS phase (shell) and the unstained PVAc phase (core). It can also be seen that the bright PVAc phase was wrapped by the dark PS protuberances, clearly implying that the prepared latex particles were of an inverted core-shell morphology containing PVAc-based cores and PS shells. From the DSC curve, shown in Figure 1d, two distinct temperature transition peaks can be found; one was the *T_g_* of the PVAc core in the low-temperature region and another was in the high-temperature region of the PS shell, manifesting that the prepared latex particles clearly had a phase-separated structure. This result clearly provides the evidence for the existence of the core-shell structure. Taken together, it can be concluded that the inverted core-shell structured PVAc-AN/PS latex particles with a phase-separated structure have been successfully synthesized by using AN as grafting unit.

### 3.2. Morphological Evolution of PVAc-AN/PS Latex Particle

**Figure 2:**
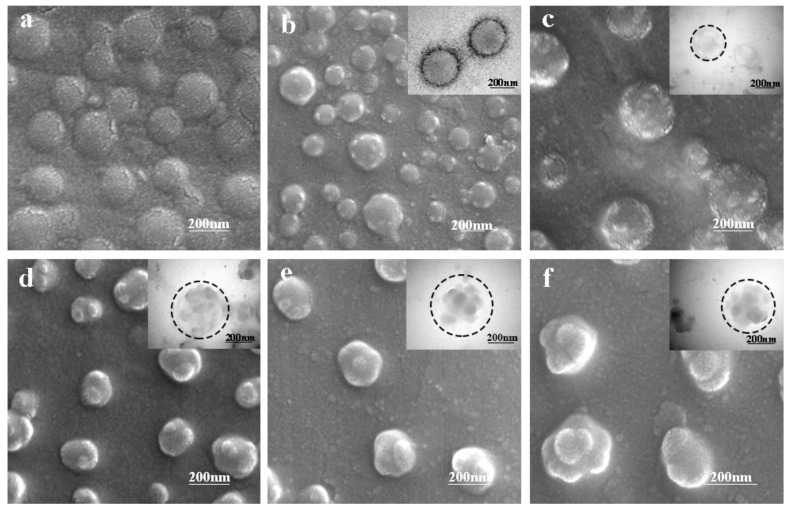
SEM and TEM images of the composite emulsion with different shell growth degrees: (**a**) PVAc core; (**b**) 30 min; (**c**) 60 min; (**d**) 90 min; (**e**) 120 min; (**f**) final state.

In general, the formation of core-shell latex particles was a layer-by-layer growth during the semi-continuous seeded emulsion polymerization, in which the initial seeds were gradually transformed into a larger ball-like core and then the shell layer underwent a gradual coating growth process by continuously adding the shell monomer. Therefore, the coating growth of the shell layer played a decisive role in the formation of the core-shell structure. As presented in Figure 1, the final morphology of the PVAc-AN/PS inverted core-shell particle was obviously a strawberry-like structure when AN was introduced into the reaction system to generate grafting sites. However, the actual mechanism through which the reaction of AN monomer affects the particle morphology and the corresponding morphological transformation process was vague. To reveal these puzzles, it is necessary to explore the dynamic transition process of the latex particle morphology during emulsion polymerization. The characteristic morphologies of the prepared inverted core-shell latex particles at different polymerization times are shown in Figure 2. Combining the microscopic images of SEM and the inserted TEM images, the inverted core-shell structure of PVAc-AN/PS latex particles can again be confirmed. For PVAc cores, the smooth spherical shape can be observed, as shown in Figure 2a. In the second stage, the St monomers were continuously added after the active grafting points on the core surface were generated by AN monomer. Figure 2b presented the latex particles with small PS protuberances after adding St at a rate of 0.2 mL/min for 30 min. The PS protuberances on the core surface displayed larger spacings and the latex particle appeared as a “hawthorn”-like structure at this stage of polymerization. With the continuous addition of shell monomers, the “peeled pomegranate”-like particles were obtained, as shown in Figure 2c. In Figure 2d, the number of bulges on the core surface decreased but the size of PS protuberances increased significantly when the shell growth polymerization extended to 90 min, manifesting that the contiguous bulges on the core surface started to merge with each other at this stage. At this stage, the morphology of latex particles can be considered as the strawberry-like type. Further increasing the number of shell monomers and prolonging the reaction time caused the strawberry-like morphology of latex particles to show only a limited change. On the other hand, the volume of the bulges was increased, as shown in Figure 2e,f. Moreover, it should be noted that the monomers, VAc and St, in the current reaction were nonionic and non-polar, signifying that the combination of PS bulges and PVAc core did not originate from electrostatic adsorption. Hence, it can be reasonably considered that the results offered strong evidence for the function of AN-induced grafting points and the position of PS protuberances can be controlled by the initial distribution of AN-based active points. 

The results of the dynamic morphology obtained from electron microscopy indicated that the morphology of PVAc-AN/PS inverted core-shell latex particles could be tuned by the polymerization time of shell growth and the evolution of particle morphology was a continuous and gradual process, manifesting that the latex particles can exhibit different forms at different shell growth stages. In summary, the thermodynamic non-equilibrium inverted core-shell structured latex particles with a unique morphology were successfully constructed, providing a strong guarantee for the production of fine PVAc-based structured particles with controllable morphologies.

### 3.3. Effect of Shell Growth on the Latex Particle Structure

**Figure 3:**
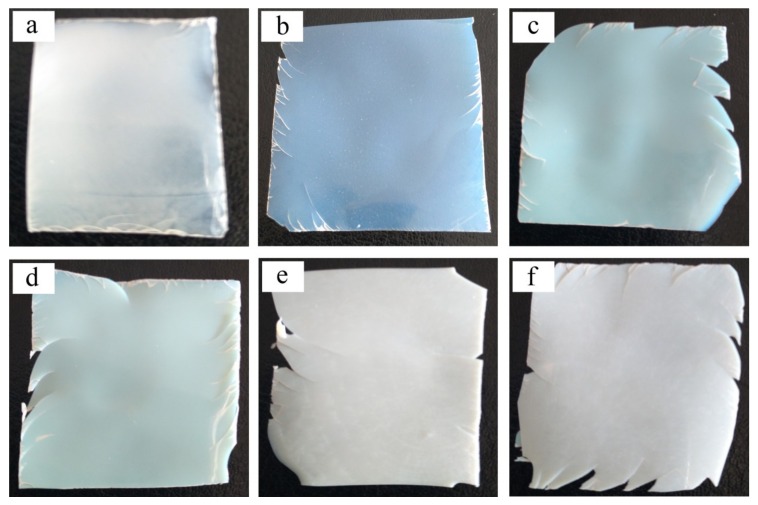
Photographs of latex films with different shell growth degrees: (**a**) PVAc core; (**b**) 30; (**c**) 60; (**d**) 90; (**e**) 120 min; (**f**) final state.

As it is well known, the film-forming process of the core-shell structured emulsion is mainly controlled by the shell polymer, leading to the fact that the performance of the latex film is determined by the properties of the shell polymer. Figure 3 presents the photographs of latex films of the inverted core-shell emulsion prepared at different shell polymerization times. It can be seen from Figure 3 that the emulsion samples with different degrees of shell growth can all be transformed to films at room temperature. In Figure 3a, the surface of the latex film obtained from PVAc core emulsion was smooth and exhibited flexibility due to the absence of a rigid PS phase. It can be seen from Figure 3b,c that the latex films were white and translucent. This was because when the degree of shell growth was low, the latex particles contained less rigid shell polymer. This can lead to a faster micro-configuration of polymer chains, resulting in latex films that have a certain transparency. By increasing the shell growth degree, more rigid shell polymer participated in the film formation process, thus the ability for the mobility and reorganization of polymer chains were weakened. As a result, the transmittance of latex films decreased and the films appeared to be milky white opaque in color, see Figure 3e,f. Furthermore, since PS was rigid with a high *T_g_*, the brittleness of latex films became more obvious upon increasing the loading amount of PS.

**Figure 4:**
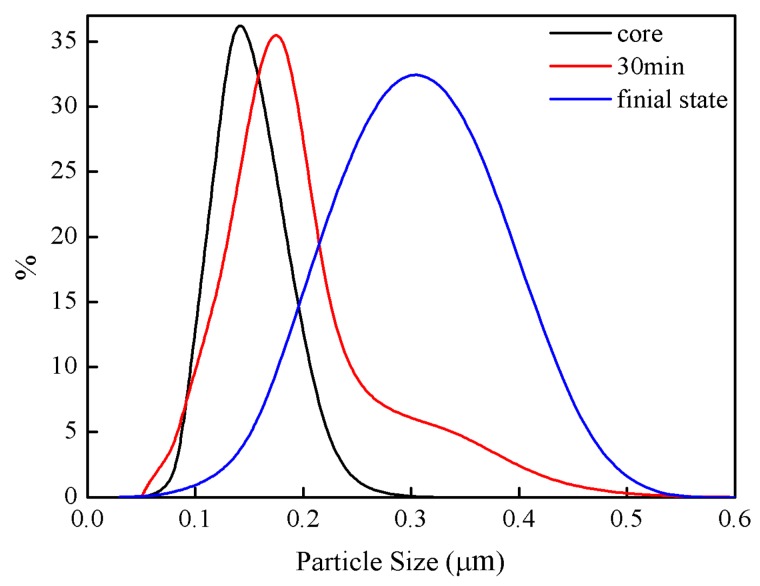
Particle size and its distribution of PVAc-AN/PS latex particles.

The particle size and its distribution of obtained PVAc-AN/PS latex samples at different reaction stages were measured by DLS, see Figure 4. According to the preparation process, the formation of inverted core-shell latex particles was a coating process in which PVAc core was gradually wrapped by PS-based shell protuberance. Accordingly, the particle size can be dependent on the thickness of the PS shell at a constant core composition. As shown in Figure 4, the size of the PVAc core was about 140 nm with a narrow distribution, indicating a uniform PVAc core latex size. When the shell polymerization time was 30 min, the particle size increased to 160 nm and had a narrow distribution. Moreover, the final particle diameter after shell monomer polymerization was approximately 320 nm and its size distribution curve displayed a relatively wide distribution. The size of the prepared PVAc-AN/PS latex particles gradually increased upon increasing the shell polymerization time, implying that the prolonged polymerization time could promote the polymerization of shell monomer. The increased size of core-shell latex particles upon extending the polymerization time presented sufficient evidence for the proposed coating growth of the PS shell during emulsion polymerization. Combined with the microscopically dynamic morphological results, see Figure 2, it can be confirmed that the formation process of PVAc-AN/PS inverted core-shell structured latexes was gradient growth.

**Figure 5:**
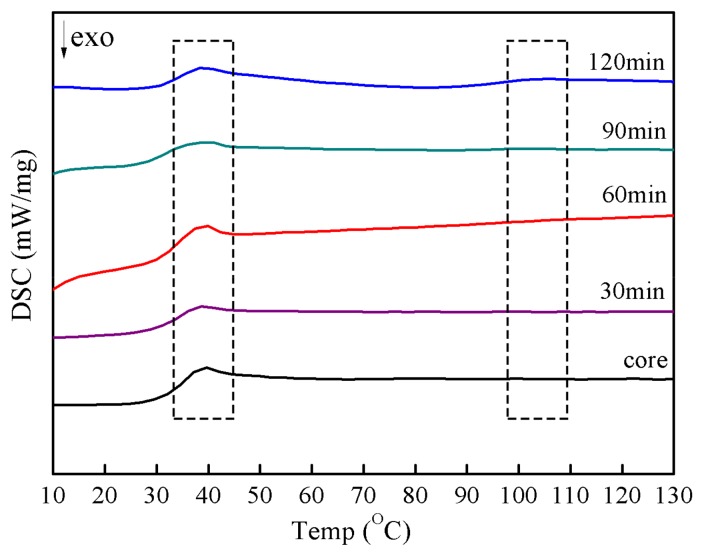
DSC curves of the composite emulsions with different shell polymerization times.

To illustrate the effect of shell growth on the morphology of the fabricated latex particles, the phase structure of latex particles was investigated by DSC, which is usually used for characterizing heterogeneous polymeric systems to unveil detailed information of the thermal properties of the polymer material, such as the glass transition temperature (*T_g_*) [25,26]. Generally, the phase-separated structure, that is, when there were two or more *T_g_* transitions during the DSC measurement, could be considered as one of the typical features for polymer-based core-shell morphology. Figure 5 displays the DSC curves of the composite latex films obtained at different shell polymerization times. In Figure 5, two distinct temperature transitions could be found when the shell polymerization time was 90 and 120 min, corresponding to the *T_g_*s of the low-temperature PVAc core polymer (38 °C) and high-temperature PS shell polymer (106 °C), respectively. The appearance of the two *T_g_*s on the curves indicated that the prepared latex particles evidently had a phase-separated structure, which was a sign for core-shell structured particles [27,28]. It could also be observed from Table 2 that the experimental *T_g_*s of the core polymer and shell polymer were in line with the ones expected according to our latex recipes (30 °C and 100 °C). Furthermore, the *T_g_* transition in the high-temperature region showed a clear gradual change in trend. When the shell polymerization time was longer than 60 min, the obtained latex particles displayed a clear baseline deflection; however, the deflection was not obvious before 60 min. With regard to the process of shell coating growth, it can be concluded that the structure of latex particles transformed from single phase to a phase-separated structure during polymerization and typically inverted core-shell structured particles were formed when the shell coating growth time was above 60 min under the current experimental conditions. This result also provided reasonable support to the fact that the morphological evolution of latex particles was controlled under the shell coating growth process.

**Figure 6:**
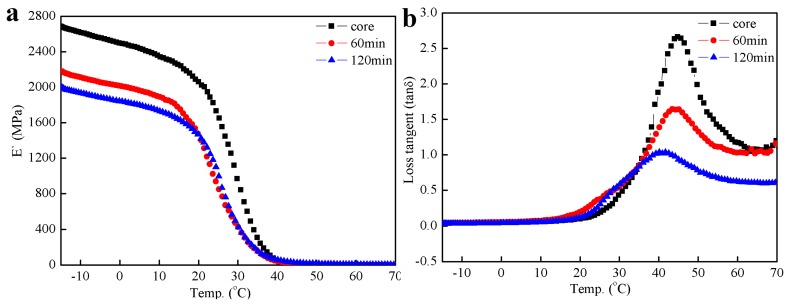
DMA curves of the composite emulsions with different shell polymerization times; (**a**) storage modulus, E′(MPa), (**b**) loss factor, tan δ.

Figure 6 presented DMA curves of the prepared inverted core-shell structured latex particles with the different shell polymerization time. In Figure 6a, the storage modulus of the PVAc-AN/PS latex films significantly decreased at the viscoelastic transition point of the shell polymer. Upon increasing the shell polymerization time, the polymer *T_g_* and the hardness of chain segments increased significantly. Accordingly, the brittleness of latex films was increased when the length and angle of the movable bond in the polymer chains were reduced, resulting in the deterioration of the viscoelastic deformation resistance caused by heat resistance. Therefore, compared to PVAc-based core latex film, the storage modulus of the structured latex films decreased when the polymerization time of the St shell monomer was extended to more than 60 min. From Figure 6b, it can be seen that, at different shell polymerization times, the corresponding temperature and peak width of the loss tangent of the latex films were similar and the peak height was different, manifesting that the composition of the shell polymer in the latex particles was the same. When the shell polymerization time was increased, that is, the shell content in the latex films increased, the proportion of the rigid shell polymer in the latex film increased, resulting in a decrease in the ratio of loss modulus to storage modulus, or, in other words, the loss tangent decreased as the shell polymerization time increased.

**Figure 7:**
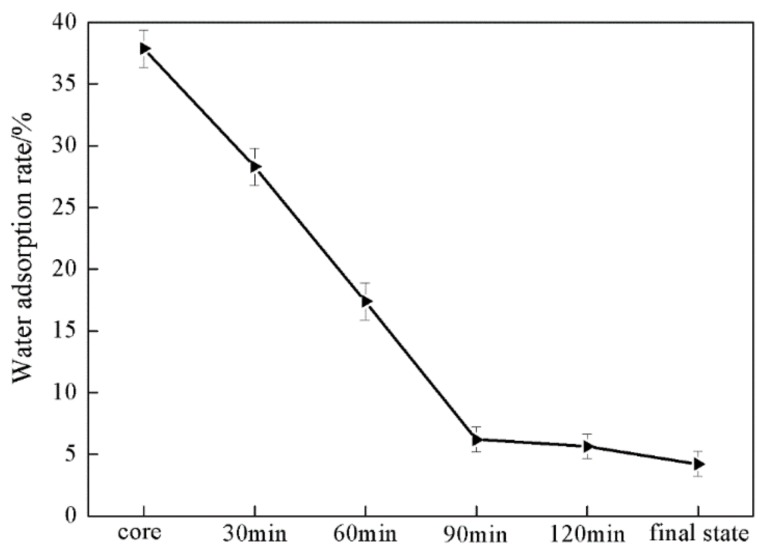
Effect of shell polymerization time on the water adsorption of latex films.

Figure 7 displayed the water absorption of PVAc-AN/PS inverted core-shell structured latex films at different reaction stages. The film obtained from PVAc core latex showed strong water absorption, which could be related to their inherent polar nature and the partial hydrolysis of ester groups in the PVAc chains. It can be seen in Figure 7 that the water absorption of prepared latex films gradually decreased with increasing shell growth time. After the shell growth time exceeded 90 min, the water absorption reached a plateau at approximately 5%. This result can be attributed to the increased coating degree of the PS shell layer onto the PVAc cores. During the process of shell coating growth, the formed protuberances of PS chains could form a protective layer on the surface of PVAc cores, preventing PVAc chains from the penetration and plastification effect enabled by water molecules, thereby reducing the damage of PVAc chains under water. Notably, the varying trend in the water absorption of latex films was consistent with the morphological evolution of latex particles at different shell growth polymerization times, clearly indicating that the morphology of the latex particles played a large role in determining its properties and that the increased coating of PS protuberance exhibited a positive impact on the water-resistant properties of PVAc-based latex films.

## 4. Formation Mechanism

**Figure 8:**
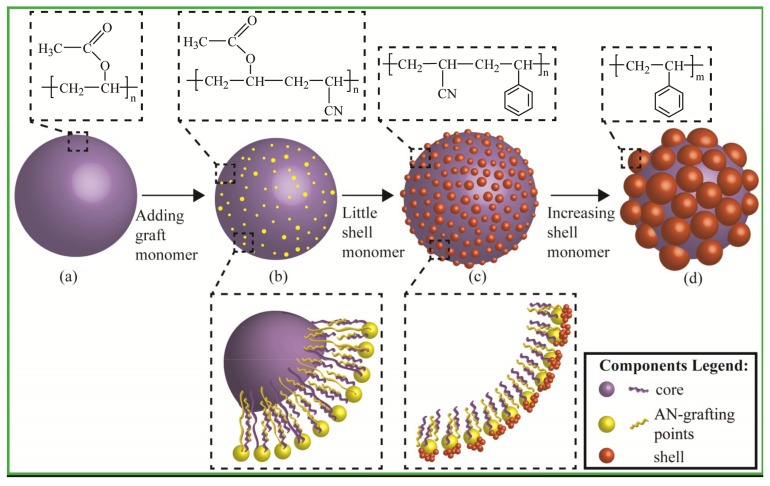
Schematic illustration for fabrication of PVAc-AN/PS inverted core-shell structured latex particles mediated by surface grafting points on PVAc-based core. (**a**) PVAc core; (**b**) core with active points; (**c**) core/shell with “peeled pomegranate”; (**d**) core/shell with strawberry type.

For the design and fabrication of core-shell structured latex particles with multiple components, an appropriate reactivity ratio of reactive monomers for polymerization and a knowledge on how to stabilize the particle structure after preparation are the prerequisites for the formation of different particle morphologies. When VAc and St monomers were directly copolymerized, they could be separated with self-polymerization processes rather than forming a core-shell structure. On the other hand, the large difference in the reactivity ratio between VAc and St and the hydrophobic feature of PS also lead to difficulties in forming a core-shell structure with internal PVAc cores and external PS shells. Hence, AN that can copolymerize with VAc and St was used as a grafting monomer to reduce the difference in the reactivity ratio between VAc and St, thereby achieving stable emulsion polymerization for VAc and St. On the other hand, due to the existence of the grafting structure on PVAc cores, the structure of the inverted core-shell structured latex particles could be stable and the phase reversal between PVAc and PS could be restricted. The schematic illustration for the fabrication of PVAc-AN/PS inverted core-shell latex particles mediated by AN-grafting is proposed in Figure 8. During the preparation of core-shell particles, the addition of different monomers was a sequential process with two stages. First, PVAc cores were obtained by seeded emulsion polymerization, see Figure 8a. When the core emulsion polymerization was completed, the grafting monomer (AN) was added into the reaction system to form active grafting sites on the surface of PVAc cores, see Figure 8b. As the activity of the double bond in the AN molecular structure was relatively strong when compared with previous structural analysis of latex particles, see Figure 1, it can be reasonably inferred that AN grafting active sites could be formed on the surface of PVAc cores, which played a key role in providing a transitional intermediary for the polymerization of shell monomers. When grafting points were generated on the surface of PVAc cores, the active points could initiate the polymerization of St monomer once shell monomer was added, see Figure 8c. In the process of shell polymerization, AN-grafting sites also acted as a targeting location, signifying that the self-polymerization of St monomer to form PS particles could be restricted and transformed to the coating growth on the surface of PVAc cores. Finally, inverted core-shell latex particles with PVAc as cores and PS as shells were obtained at the end of polymerization, see Figure 8d. In other words, although the location of the St monomer polymerization was transformed to the core surface owing to AN-induced graft points, the polymerization of St monomer was still self-polymerization. Therefore, based on the semi-continuous seeded emulsion polymerization process and the generation of active grafting sites on the surface of PVAc cores, the PVAc-AN/PS inverted core-shell structured latex with controllable latex morphologies could be fabricated.

**Figure 9:**
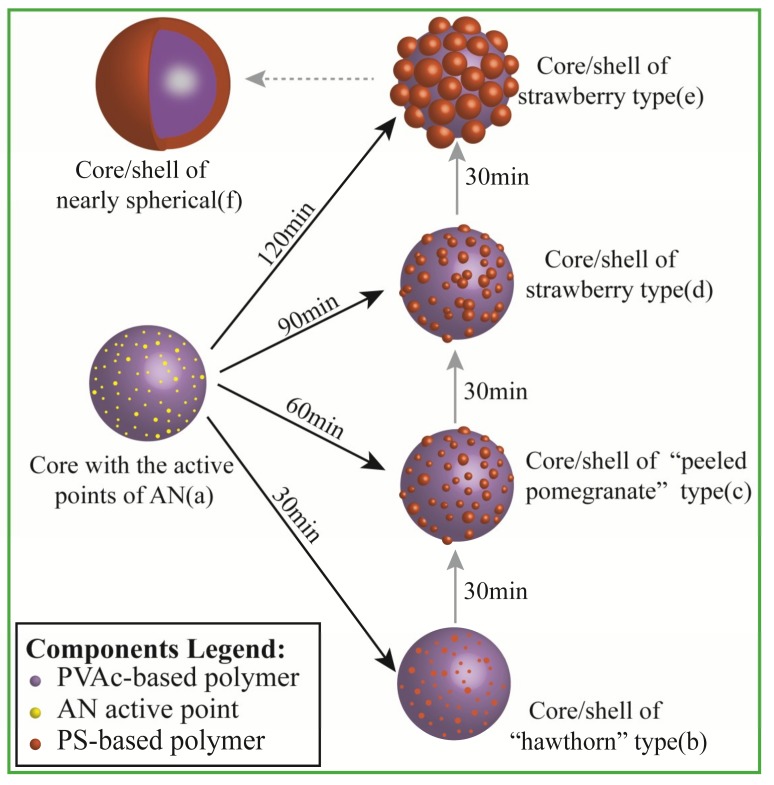
Formation mechanism and morphological evolution of PVAc-AN/PS inverted core-shell structured latex particles.

For structured latex particle morphology, a ball-like particle was the most readily available form based on the minimum energy principle. In the process of obtaining ball-like particles, interfacial interaction between an aqueous and organic phase was the main driving force. Although the ball-like morphology of PVAc-AN/PS inverted core-shell latex particles was hard to achieve, it was easy to form a series of distinctive morphologies during the shell formation, such as hawthorn-like, peeled pomegranate-like, and strawberry-like, as shown in Figure 2. The formation mechanism of these thermodynamic non-equilibrium latex particles was distinctly different compared to the ball-like particles. Therefore, on the basis of the experimental results above, the following mechanism of the morphological development of latex particles was proposed in Figure 9. As depicted in Figure 9a, under the given polymerization condition, graft monomers (AN) approached the PVAc core surface to form active grafting sites which could initiate St polymerization, as mentioned in Figure 8. The number of AN-grafting sites hence affected the final morphology of the latex particles to some extent. More grafting sites can increase the chance for initiating St polymerization on the surface of PVAc cores, enabling an increased number of PS protuberances and enlarging the shell coating degree. 

The *T_g_* of PS (100 °C) is higher than that of the reaction temperature (80 °C), leading to the fact that the movement of PS chain segments was restricted throughout the whole process of shell polymerization. Therefore, the shell polymerization of St monomer could be considered as in situ coating growth. The chain growth of PS occurred around the AN-grafting sites, and the broadened coating domain of single PS protuberance to PVAc core was also restricted. During the early stage of shell polymerization, a large number of tiny PS protuberances were formed on the core surface and the morphology of latex particles was similar to “hawthorn fruit”, see Figure 9b. As the reaction continued, the volume of these PS bulges gradually increased, the spacing between adjacent PS bulges became smaller, and the latex particles presented “peeled pomegranate”-like morphology, see Figure 9c. At this stage, the coating growth of the PS shell was confined to the initial position of the AN-grafting sites and the thermal motion of PS chains to reduce the interfacial energy was greatly hampered by its own glass state. Thus, the morphological development of latex particles depended on the gradual increase in PS protuberances. Upon further enlarging the size of PS protuberances, the adjacent protuberances were in contact with each other and the merging between these bulges unavoidably occurred in the area of contact. The merging between protuberances was achieved by the movement of the bond distance and bond angle of PS chain segments and could be further deepened as long as the shell polymerization continued. The strawberry-like latex particle depicted in Figure 9d was the result of bulges merging. The combined protuberances gradually formed an obvious strawberry-like structure following the continuation of shell polymerization, see Figure 9e. Throughout the morphological evolution of PVAc-AN/PS inverted core-shell latex particles, it could be found that the morphology of the latex particles tended to be ball-like. Therefore, it can be reasonably deduced that if the process of shell growth continued, the ball-like PVAc-AN/PS latex particles might be formed, which was a fully coated inverted core-shell structure, see Figure 9f. Taken together, it can be concluded that the synthesis of the PVAc-AN/PS inverted core-shell structured latex could be inferred as in situ coating growth of the PS shell onto the PVAc core surface, whereas the fundamental polymerization of PS was unchanged and was still self-polymerization. The introduction of the active grafting sites only changed the polymerization site of PS, which caused the self-polymerization of PS to transform into the coating growth of PS onto the PVAc cores. It was noteworthy that the morphology of the prepared latex particles had a clear dynamic evolution during the process of shell polymerization. The morphology also affected the performance of latex particles; not only did the water absorption of latex films clearly drop but latex particles showed an obvious phase-separated structure. In summary, the preparation of such latex particles provided a controllable path for the fabrication of specific morphological core-shell latex particles.

## 5. Conclusions

Thermodynamic non-equilibrium PVAc-AN/PS inverted core-shell structured latex particles with controllable morphologies and surface topographies were successfully synthesized via semi-continuous seeded emulsion polymerization. The construction of AN-active grafting points not only changed the self-polymerization sites of the shell monomer but also laid the foundation for the stable combination of hydrophilic PVAc core and hydrophobic PS shell. The dynamic morphological evolution of the prepared latex particles was easily adjusted by altering in situ coating growth of the PS shell and characteristic morphologies, transferring from hawthorn-like, to peeled pomegranate-like, and, finally, to strawberry-like, could be formed. Furthermore, the morphological effect of PVAc-AN/PS latex particles could be specifically reflected in the film properties. By increasing the coating degree of the shell polymer on PVAc cores, the structure of latex films gradually changed from the single-phase structure to a two-phase-separated structure, and the thermomechanical properties of latex film decreased accordingly. The water absorption of latex films decreased sharply with increasing shell polymerization degree and the lowest value was less than 5%. The fabricating strategy may offer a number of possibilities for the controllable design of heterogeneous core-shell morphologies for a variety of applications.

## Figures and Tables

**Table 1:** Recipes and reaction conditions for synthesizing polyvinyl acetate (PVAc)-acrylonitrile (AN)/polystyrene (PS) latexes.

Codes		CS-1	CS-2	CS-3	CS-4	CS-5
Seed latex	Temperature/°C	60	60	60	60	60
	m(Water)/g	70	70	70	70	70
	m (VAc)/g	16	16	16	16	16
	m(PCA078)/g	2.5	2.5	2.5	2.5	2.5
	m(PCA507)/g	2.5	2.5	2.5	2.5
	m(NaHCO_3_)/g	0.3	0.3	0.3	0.3	0.3
	m(APS)/g	0.1	0.1	0.1	0.1	0.1
Core-shell latex	Temperature/°C	80	80	80	80	80
	m (VAc)/g	54	54	54	54	54
	m (St)/g	30	30	30	30	30
	m (AN)/g	2	2	2	2	2
	m(APS)/m(water)/(g·g^−1^)	0.25/20	0.25/20	0.25/20	0.25/20	0.25/20
	The time of shell polymerization/min	30	60	90	120	final state

**Table 2:** Designed and experimental glass transition temperature (*T_g_*) of film samples.

Codes	*T_g_* (Core Polymer) (°C)		*T_g_* (Core Polymer) (°C)	
	Designed *T_g_*	Experimental *T_g_*	Designed *T_g_*	Experimental *T_g_*
core	30	38	/	/
30 min	30	38	100	/
60 min	30	38	100	/
90 min	30	38	100	106
120 min	30	38	100	106

## References

[1] Ma J.Z., Liu Y.H., Bao Y., Liu J.L., Zhang J. (2013). Research advances in polymer emulsion based on “core-shell” structure particle design. Adv. Colloid Interface Sci..

[2] Ferguson C.J., Russell G.T., Gilbert R.G. (2002). Modelling secondary particle formation in emulsion polymerisation: Application to making core-shell morphologies. Polymer.

[3] Pérez-Carrillo L.A., Puca M., Rabelero M., Meza K.E., Puig J.E., Mendizábal E., López-Serrano F., López R.G. (2007). Effect of particle size on the mechanical properties of polystyrene and poly(butyl acrylate) core/shell polymers. Polymer.

[4] Sahiner N., Butun S., Ilgin P. (2011). Hydrogel particles with core shell morphology for versatile applications: Environmental, biomedical and catalysis. Colloids Surf. A Physicochem. Eng. Asp..

[5] Schroffenegger M., Reimhult E. (2018). Thermoresponsive Core-Shell Nanoparticles: Does Core Size Matter?. Materials.

[6] Xu M., Liu J., Xu X., Liu S., Peterka F., Ren Y., Zhu X. (2018). Synthesis and Comparative Biological Properties of Ag-PEG Nanoparticles with Tunable Morphologies from Janus to Multi-Core Shell Structure. Materials.

[7] Pei X., Zhai K., Tan Y., Xu K., Lu C., Wang P., Wang T., Chen C., Tao Y., Dai L. (2017). Synthesis of monodisperse starch-polystyrene core-shell nanoparticles via seeded emulsion polymerization without stabilizer. Polymer.

[8] Lee C.F. (2005). Effects of surfactants on the morphology of composite polymer particles produced by two-stage seeded emulsion polymerization. J. Polym. Sci. Part A Polym. Chem..

[9] Klein M.K., Klinkenberg N., Schuetter S., Saenger N., Pfleiderer P., Zumbusch A. (2015). PMMA/PMMA core-shell particles with ellipsoidal, fluorescent cores: Accessing rotational dynamics. Langmuir.

[10] Carrà S., Sliepcevich A., Canevarolo A., Carrà S. (2005). Grafting and adsorption of poly(vinyl) alcohol in vinyl acetate emulsion polymerization. Polymer.

[11] Zhang X., Bai L., Lou C., Chen X., Jia Z., Gu J., Li Z. (2016). Fabrication and morphological evolution of inverse core/shell structural latex particles of poly(vinyl acetate)/polystyrene by maleic anhydride grafting. Colloid Polym. Sci..

[12] Bai L., Huan S., Zhang X., Jia Z., Gu J., Li Z. (2017). Rational design and synthesis of transition layer-mediated structured latex particles with poly(vinyl acetate) cores and poly(styrene) shells. Colloid Polym. Sci..

[13] Lin C.L., Chiu W.Y., Lee C.F. (2005). Thermal/pH-sensitive core-shell copolymer latex and its potential for targeting drug carrier application. Polymer.

[14] Aguirre M., Paulis M., Barrado M., Iturrondobeitia M., Okariz A., Guraya T., Ibarretxe J., Leiza J.R. (2014). Evolution of particle morphology during the synthesis of hybrid acrylic/CeO_2_ nanocomposites by miniemulsion polymerization. J. Polym. Sci. Part A Polym. Chem..

[15] Xuan S., Wang Y.J., Leung K.C., Shu K., August R.V., Re V., Recei M., October V. (2008). Synthesis of Fe_3_O_4_@Polyaniline Core/Shell Microspheres with Well-Defined Blackberry-Like Morphology. J. Phys. Chem. C.

[16] Song T., Liu T., Yang X., Bai F. (2015). Raspberry-like particles via the heterocoagulated reaction between reactive epoxy and amino groups. Colloids Surf. A Physicochem. Eng. Asp..

[17] Bonnefond A., Pereira Gomes C., de la Cal J.C., Leiza J.R. (2017). Surfactant-free poly(methyl methacrylate)/poly(vinylamine) (PMMA/PVAm) amphiphilic core-shell polymer particles. Colloid Polym. Sci..

[18] McKenzie A., Hoskins R., Swift T., Grant C., Rimmer S. (2017). Core (Polystyrene)-Shell [Poly(glycerol monomethacrylate)] Particles. ACS Appl. Mater. Interfaces.

[19] Ferguson C.J., Russell G.T., Gilbert R.G. (2003). Synthesis of latices with hydrophobic cores and poly(vinyl acetate) shells. 2. Use of poly(vinyl acetate) seeds. Polymer.

[20] Ferguson C.J., Russell G.T., Gilbert R.G. (2002). Synthesis of latices with polystyrene cores and poly(vinyl acetate) shells. 1. Use of polystyrene seeds. Polymer.

[21] Aizpurua I., Amalvy J.I., Barandiaran M.J. (2000). Influence of the Polymeric Hidrophobe on the Kinetics of Vinyl Acetate Miniemulsion Polymerization. Colloids Surf. A Physicochem. Eng. Asp..

[22] Chiozza F., Pizzo B. (2016). Innovation in poly(vinyl acetate) water resistant D3 glues used in wood industry. Int. J. Adhes. Adhes..

[23] Joensson J.E.L., Hassander H., Jansson L.H., Toernell B. (1991). Morphology of two-phase polystyrene/poly(methyl methacrylate) latex particles prepared under different polymerization conditions. Macromolecules.

[24] Ramli R.A., Laftah W.A., Hashim S. (2013). Core–shell polymers: A review. RSC Adv..

[25] Karlsson O.J., Hassander H., Colombini D. (2003). The effect of first-stage polymer Tg on the morphology and thermomechanical properties of structured polymer latex particles. C. R. Chim..

[26] Stubbs J.M., Sundberg D.C. (2005). Measuring the extent of phase separation during polymerization of composite latex particles using modulated temperature DSC. J. Polym. Sci. Part B Polym. Phys..

[27] Stubbs J.M., Sundberg D.C. (2003). Fundamental studies on morphology control for latex systems with application to waterborne coatings: The effect of polymer radical mobility in latex particles during polymerization. J. Coat. Technol..

[28] Hergeth W.D., Bittrich H.J., Eichhorn F., Schlenker S., Schmutzler K., Steinau U.J. (1989). Polymerizations in the presence of seeds: 5. Core-shell structure of two-stage emulsion polymers. Polymer.

